# Protein Homeostasis Defects of Alanine-Glyoxylate Aminotransferase: New Therapeutic Strategies in Primary Hyperoxaluria Type I

**DOI:** 10.1155/2013/687658

**Published:** 2013-07-16

**Authors:** Angel L. Pey, Armando Albert, Eduardo Salido

**Affiliations:** ^1^Departamento de Química-Física, Facultad de Ciencias, Universidad de Granada, Avenida Fuentenueva s/n, 18071 Granada, Spain; ^2^Departamento de Cristalografía y Biología Estructural, Instituto de Química Física “Rocasolano”, Consejo Superior de Investigaciones Científicas, Serrano 119, 28006 Madrid, Spain; ^3^Centre for Biomedical Research on Rare Diseases (CIBERER), University Hospital of the Canary Islands, and CIBICAN, University of La Laguna, 38320 Tenerife, Spain

## Abstract

Alanine-glyoxylate aminotransferase catalyzes the transamination between L-alanine and glyoxylate to produce pyruvate and glycine using pyridoxal 5′-phosphate (PLP) as cofactor. Human alanine-glyoxylate aminotransferase is a peroxisomal enzyme expressed in the hepatocytes, the main site of glyoxylate detoxification. Its deficit causes primary hyperoxaluria type I, a rare but severe inborn error of metabolism. Single amino acid changes are the main type of mutation causing this disease, and considerable effort has been dedicated to the understanding of the molecular consequences of such missense mutations. In this review, we summarize the role of protein homeostasis in the basic mechanisms of primary hyperoxaluria. Intrinsic physicochemical properties of polypeptide chains such as thermodynamic stability, folding, unfolding, and misfolding rates as well as the interaction of different folding states with protein homeostasis networks are essential to understand this disease. The view presented has important implications for the development of new therapeutic strategies based on targeting specific elements of alanine-glyoxylate aminotransferase homeostasis.

## 1. Alanine-Glyoxylate Aminotransferase and Primary Hyperoxaluria Type I

Alanine-glyoxylate aminotransferase (AGT) is one of the aminotransferases that has raised most biomedical interest, since its deficiency causes primary hyperoxaluria type I (PH1), a rare inherited entity with unique features in terms of cellular and molecular biology of human disease. AGT, encoded by the *AGXT* gene, catalyzes the transamination between L-alanine and glyoxylate to produce pyruvate and glycine using pyridoxal 5′-phosphate (PLP) as cofactor.

As it has been the case for a number of advances in the understanding of the molecular basis of disease, the in-depth analysis of the pathogenesis of PH1 has shed light into a broader field, such as the subcellular compartmentalization of enzymes or the effect of gene modifiers on phenotype and the synergy between mutations and common genetic polymorphisms. 

### 1.1. Role of AGT in Glyoxylate Metabolism

**Figure 1:**
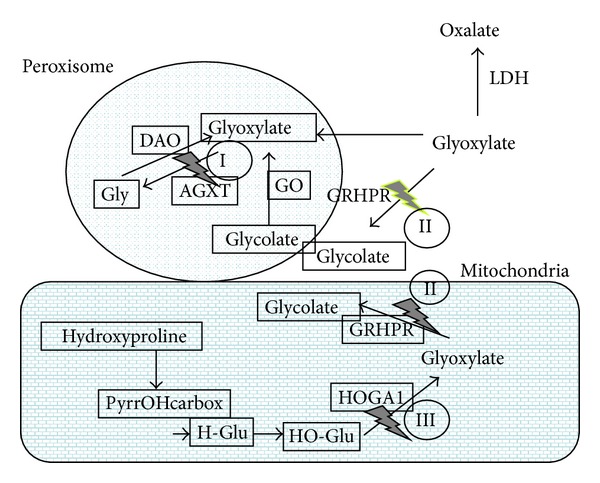
Summary of the glyoxylate metabolism in human hepatocytes. Simplified pathways involving glycine, glycolate, and hydroxyproline as the main sources of glyoxylate. Peroxisomal glyoxylate is detoxified by AGT, while mitochondrial and cytosolic glyoxylate is reduced to glycolate by GRHPR, preventing excessive oxidation to oxalate by LDH. Hydroxyproline metabolism results in the production of 4-hydroxy-2-oxoglutarate that is normally split into glyoxylate and pyruvate by HOGA1. PyrrOHcarbox=pyrroline-5-carboxylate; HGlu= 4-hydroxy-glutamate; HO-Glu=4-hydroxy-2-oxoglutarate. The three genetic defects currently known to cause PH are inherited with autosomal recessive pattern. The genes involved are alanine-glyoxylate aminotransferase (*AGXT*, at 2q37.3, MIM∗604285), for PH type I (PH1, MIM#259900), glyoxylate reductase/hydroxypyruvate reductase (GRHPR, at 9q12, MIM∗604296), for PH type II (PH2, MIM#260000), and 4-hydroxy-2-oxoglutarate aldolase (HOGA1 at 10q24.2, MIM∗613597), for PH type III (PH3, MIM#613616).

Glyoxylate is a two-carbon keto-acid of intermediary metabolism, with glycine, glyoxal, hydroxyproline, and glycolate as its best known sources in humans. Glyoxylate is readily converted into oxalate by various dehydrogenases and oxidases, including lactate dehydrogenase (LDH). Oxalate is an end product of metabolism in mammals that has to be eliminated with the urine; otherwise, it tends to precipitate as tissue-damaging calcium oxalate. The relevance of glyoxylate detoxification to human health is underscored by the deleterious consequences of inherited mutations in genes coding for key enzymes in this pathway, *AGXT* being one of them (Figure 1). Human conditions characterized by high oxalate levels in urine are known as hyperoxalurias, and their genetic forms, termed primary hyperoxalurias (PH), are due to high oxalate production by hepatocytes deficient in one of these enzymes [1–3]. PH patients have urinary excretion levels >0.5 mmoL/1.73 m^2^ per day (typically >1 mmoL/1.73 m^2^), while normal oxalate excretion is below 0.45 mmoL/1.73 m^2^.

Since LDH is abundant in the hepatocyte cytosol and vertebrates do not have a functional glyoxylate shunt capable of using glyoxylate as a substrate for the tricarboxylic acid cycle, most of the glyoxylate generated must be metabolized within organelles such as the peroxisome and mitochondria in order to limit oxalate production. To further control the levels of oxalate produced, cytosolic glyoxylate reductase (GRHPR) competes with LDH for glyoxylate, reducing it to glycolate, a highly soluble two-carbon molecule.

Glyoxylate detoxification reflects the evolutionary origins of metabolic partitioning into the various subcellular organelles [5]. Thus, the subcellular distribution of the key enzymes of the glyoxylate detoxification pathway has been under evolutionary pressure and diet must have been an important component of such pressure, since glycolate is abundant in vegetables while hydroxyproline is abundant in meat. 

Human AGT is a hepatocyte-specific enzyme that is normally located in the peroxisomes only [6], making this organelle an efficient site for detoxification of glyoxylate either imported from the cytosol or mitochondria or produced in situ by either D-amino acid oxidase (DAO) or hydroxyacid oxidase (HAO1) (glycolate oxidase), using glycine or glycolate as substrate, respectively. The peroxisome membrane is permeable to glycolate, glyoxylate, and other small hydrophilic solutes, largely through the PXMP2 channel [7]. Since AGT can tolerate high glyoxylate concentrations [8], the peroxisome, rich in AGT, plays a crucial role as glyoxylate detoxifying compartment that shields the surrounding cytoplasm from glyoxylate accumulation and secondary oxalate production.

Mitochondria also play an important role in glyoxylate metabolism [9, 10]. In humans, this role is based on their capacity to metabolize hydroxyproline [11], but in mammals with mitochondrial AGT this enzyme is also central to glyoxylate detoxification in this organelle. Collagen, containing ~15% hydroxyproline, is a major constituent of extracellular matrix and daily collagen turnover yields 300–450 mg hydroxyproline, accounting for the production of 180–240 mg glyoxylate [12, 13]. The last step of this pathway involves the cleavage of 4-hydroxy-2-oxoglutarate (2-keto-4-hydroxyglutarate) into glyoxylate and pyruvate by 4-hydroxy-2-oxoglutarate aldolase (HOGA1). The glyoxylate can then be converted to glycolate by GRHPR. 

### 1.2. Primary Hyperoxaluria Type I

PH has an estimated prevalence of 1–3 per million population and an estimated incidence rate of ~1 : 100,000 live births per year in Europe [14–16], although the exact prevalence is unknown due to underdiagnosis. The most comprehensive attempts to estimate the true incidence of the disease [16] have resulted in higher incidence rates than previously reported. Higher rates have also been found in historically isolated populations, like the Canary Islands, due to founder effect [17]. Although PH accounts for less than 1% of children in end-stage renal disease (ESRD) registries of developed countries [18], almost 10% of Kuwaiti children and 13% of Tunisian children with ESRD have been reported to suffer PH [19, 20].

PH1, caused by deficient or mistargeted AGT [21], is the most common (around 80%) and the most severe PH type, usually resulting in ESRD at some point, although with a wide range of severity. At ESRD, the buildup of oxalate in the body (known as oxalosis) quickly results in bone, heart, skin, and retinal complications. Oxalosis is a life-threatening condition, unless liver-kidney transplantation is performed [14, 17, 22]. 

The interest in PH1 prompted the cloning of *AGXT* cDNA [23, 24], using probes from the orthologous rat gene [25]. The gene has 11 exons and spans ~10 kb [26], resulting in a 1.7 kb mRNA with a coding sequence of 1,176 bp. The gene product AGT is a homodimeric protein, each 43 kDa subunit containing 392 amino acids and holding one molecule of PLP as cofactor [27]. The main N-terminal domain contains most of the catalytic active site, the cofactor-binding site, and the dimerization interface. The smaller C-terminal domain is known to interact with the peroxisomal receptor PEX5, targeting the dimer to the peroxisome. AGT, carrying a noncanonical peroxisomal targeting sequence (PTS1), is among the peroxisomal proteins with the weakest affinities for PEX5 [28]. An ancillary sequence surrounding amino acids 324–345 has been proposed to help the peroxisomal targeting of AGT [29]. A recently released crystal structure of the AGT in complex with the PTS1-binding domain of PEX5 (PDB: 3IMZ) also confirmed that residues 303–306 and 327–330 are largely buried upon binding. In fact, AGT binds to PEX5 with ~10-fold higher affinity than its PTS1 octapeptide, showing the functional role of this ancillary sequence in PEX5 binding [30].

The 3D structure of AGT (PDB: 1H0C) [27] has provided important information to better understand the function of the protein and the effect of changes in amino acid that account for a majority of the PH1 mutations (see Section 3 below). 

More than 150 mutations have been described for *AGXT*, and they have been summarized recently [31]. Missense mutations are common, followed by small insertion/deletions (indels). Wild type *AGXT* comes in two polymorphic variants, the most frequent major haplotype (refseq NM_000030) and the less frequent minor haplotype, carrying two single amino acid substitutions (p.P11L and p.I340M) among other genomic changes in strong linkage disequilibrium. Since these two polymorphisms are quite old, most of the individual PH1 mutations described are typically found in either the major or minor haplotype, but rarely in both, which is useful when searching for mutations in new PH1 cases [32]. The minor haplotype (simply defined by the refSNP rs34116584, p.Pro11Leu), with an allelic frequency of 0.1-0.2 in western countries and average heterozygosity around 0.2, does not cause PH1 by itself, but it is known to act synergistically with the deleterious effects of several common mutations [33, 34].

### 1.3. Molecular Mechanisms of Disease

*AGXT* mutations result in severe reductions of AGT enzymatic activity in the peroxisome, with a relatively wide range of residual activity, depending on the mutations present in both alleles [35]. Although AGT functions as a dimer, all the mutations described so far are related to loss of function, with recessive pattern of inheritance, without evidence of potential dominant negative effect.

Small indels are responsible for some PH1 cases due to AGT synthesis defects, most notably c.33dupC, the main mutation of the major haplotype, with the predicted consequence of early stop codon and nonsense mediated mRNA decay. We could also include in this category of synthesis defects most splicing mutations and occasional missense mutations leading to highly unstable protein that is degraded rapidly, such as p.S205P [36]. But the majority of PH1 alleles are missense mutations, with four potential molecular mechanisms involved: mitochondrial mistargeting, protein aggregation, catalytic defects, and enhanced turnover.

About a third of PH1 alleles involve the p.G170R substitution, in the minor haplotype, which is responsible for mitochondrial mistargeting of the gene product, becoming one of the best known examples of human mutations resulting in mistargeting as the main mechanism of disease [37–39]. In addition to p.G170R, p.F152I, also in the minor haplotype, was found to cause AGT mistargeting to the mitochondria instead of the peroxisome, depleting the latter organelle of its glyoxylate detoxifying capability.

The polymorphism p.P11L of the minor haplotype plays a crucial role for p.G170R to result in mitochondrial mistargeting [40], which has been associated to impaired folding efficiency to form functional dimers, therefore allowing mitochondrial import of mutant AGT [41]. 

Protein aggregation is a relatively frequent outcome of missense mutations in conformational diseases [42]. Several of the most frequent PH1 mutations of the minor haplotype, such as p.G41R [37] and p.I244T [34], are known to display protein aggregation. The p.P11L polymorphism was also found to play a crucial role for the I244T mutation to result in a conformationally unstable protein, prone to aggregation [34], while the p.G41R mutation disturbs the local interactions of the N-terminus, causing conformational instability as well [43].

Catalytic defects are a common mechanism of disease in inborn errors of metabolism involving enzyme-coding genes. Enzymatic characterization of several PH1 missense mutations (p.G82E, p.G41R, and p.F152I) have demonstrated significant alterations in their performance, including strong decreases in catalytic efficiency and reduced binding affinities for PLP and PMP cofactors [44].

Genotype-phenotype correlations have been described for some mutations of the *AGXT* gene [33, 45, 46], but the wide allelic heterogeneity limits this type of analysis to the most common mutations, unless large international registries are used. Significant environmental influences and the potential effect of genetic modifiers also play an important role to the point that siblings who share the same genotype could have very different clinical phenotypes [47].

## 2. Structure-Function Relationships of AGT

**Figure 2:**
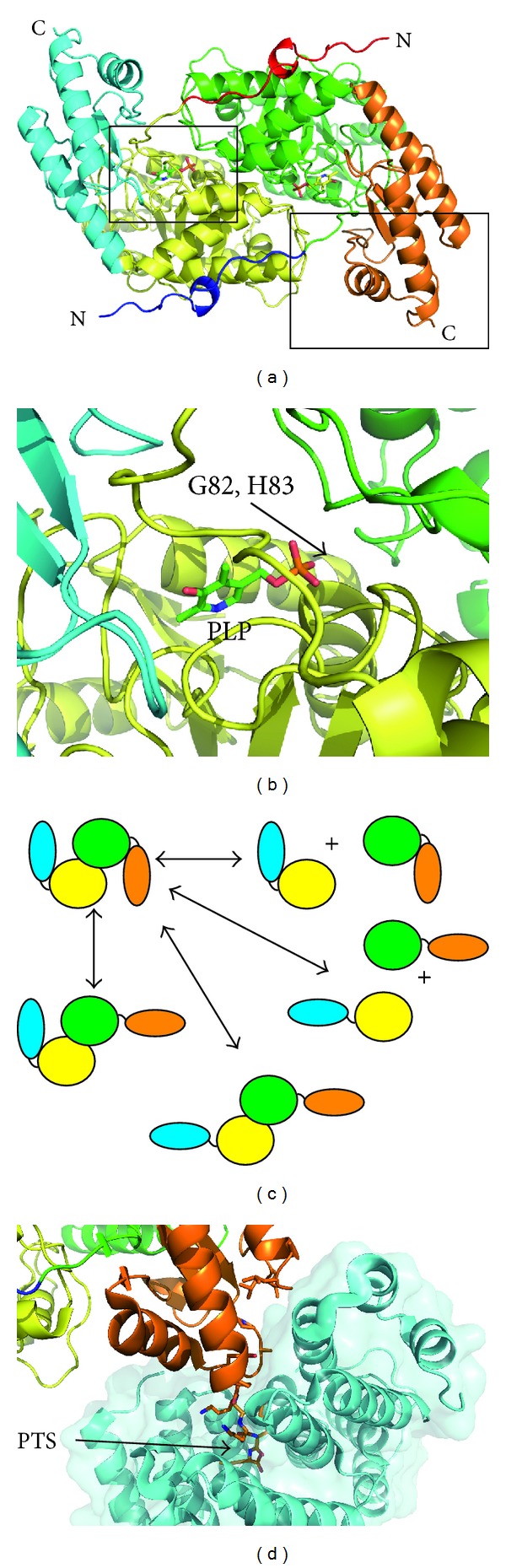
The structure of AGT. (a) A ribbon representation of the dimeric AGT structure (PDB code 1H0C) colored to highlight the domain organization. The black squares represent the zoomed sections shown in panels 1(b) and 1(d). (b) A representation of the PLP binding site; G82 and H83 are in the vicinity of the cofactor. (c) A schematic representation of the possible “almost folded” AGT intermediates. (d) A representation of the AGT Pep5x intermolecular interface. The molecular surface representation of Pex5p is also displayed and colored in cyan. Those AGT residues interacting with Pep5x are displayed in a stick representation.

The crystal structure of AGT was solved at atomic resolution [27] to gain insights on the molecular and structural basis of the disease. AGT is a homodimer with each protomer folded into a large N-terminal domain (residues 22–282) and a smaller C-terminal domain (283–392). Most of the contacts within the dimer involve the large N-terminal domain. Besides, a long unstructured N-terminal tail (residues 1–21) grabs the subunits within the dimer (Figure 2(a)). This structure provided valuable information about the enzyme functioning and a framework to map the mutations of the AGT gene. This analysis showed that they are almost randomly scattered over the entire three-dimensional structure of the enzyme [30], making it difficult to establish general rules on the molecular consequences of these variants. However, the available structural information resolves central questions on the knowledge about the basis of the disease.

The effect of mutations affecting the enzymatic properties of AGT can be rationalized in structural terms. p.G82E and p.H83R are well characterized to produce catalytic defects [8, 48]. The analysis of the structure reveals that both of them cluster in the vicinity of the active site. These changes involve bulkier side chains that hinder either cofactor or substrate binding (Figure 2(b)). By contrast, the attempts to rationalize the effect of variants leading to protein aggregation in terms of the structure have been unsuccessful since it is likely that either kinetic and/or thermodynamic changes are the main mechanism involved [48]. The modeling of these mutations on the structure reveals no significant changes since they often involve either conservative changes or solvent accessible residues. Consequently, it is not expected to induce large predictable conformational changes in AGT structure. This is well illustrated by the structural analysis of the G170R variant, which resulted in a nearly identical structure to the wild type [49]. In addition, the expression of many of these mutations yields unstable aggregated and/or partially unfolded products that cannot be crystallized. 

The three-dimensional structure of the complex between the ring chaperonin GroEL and AGT-LTM variant provided evidence that the mutated enzyme is able to form nonnative folding intermediates that interact with the chaperone [50]. These intermediates consist of an “open” version of AGT protomer in which the small and the large domains are correctly folded (Figure 2(c)). Such intermediates may be prone to protein aggregation but they constitute a promising target for pharmacological chaperones.

The crystal structure of the AGT in complex with the PTS1-binding domain of Pex5p was determined to shed light into the mechanism of AGT import into peroxisomes [30]. The complex displays Pex5p-AGT-AGT-Pex5p stoichiometry and confirms that dimeric and perfectly folded AGT interacts with the receptor. On the AGT side, the complex buries completely those residues forming the PTS1 and some constituting the smaller C-terminal domain (Figure 2(d)). This suggests that those mutations affecting the surface properties of the interaction area will affect the import to peroxisomes.

## 3. Biophysical, Biochemical, and Cell Biology Approaches to Study AGT Deficiency and Protein Homeostasis Defects

**Figure 3:**
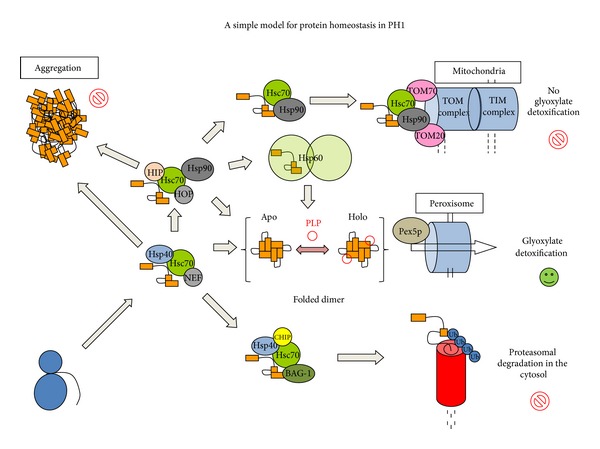
A simple scheme representing potentially important checkpoints in the folding and misfolding of human AGT. After ribosomal synthesis, the AGT monomer is maintained in a partially folded and soluble state upon interaction with Hsp40 chaperones allowing its engagement to the Hsp70 machinery. Correct folding may proceed through the transfer of the partially folded polypeptide to the Hsp90 and Hsp60 machineries leading to the folded holo-AGT dimer and peroxisomal import through the Pex5p import machinery. However, PH1 causing mutants to show enhanced interactions with Hsp70, Hsp60, and Hsp90 chaperone systems which may (i) delay correct folding eventually causing AGT aggregation; (ii) allow engaging the proteasomal degradation machinery mediated by CHIP and BAG-1 proteins; (iii) allow Hsp70/Hsp90 mediated presentation to the mitochondrial import machinery via TOM20 or TOM70 receptors. For further details and references, see the main text.

Protein homeostasis controls the functional properties of proteins by minimizing the presence of misfolded protein states that may be damaging to cellular function [51, 52]. A complex and highly interacting and regulated network of pathways, involving over 800 different proteins, is in charge of protein homeostasis [53], regulating protein synthesis, folding, trafficking, and degradation [51, 52]. Protein homeostasis defects are associated with aging and disorders of protein folding, including metabolic diseases, cancer, and neurodegenerative diseases [51, 52, 54]. In the context of protein homeostasis, intrinsic physicochemical properties of polypeptide chains such as thermodynamic stability, folding, unfolding, and misfolding rates as well as the interaction of different folding states with protein homeostasis networks are essential to understand protein folding and misfolding under physiological and pathological conditions [54]. In this section, we summarize the knowledge on protein homeostasis defects in PH1 to provide a comprehensive and integrated perspective of PH1 as a folding disease. The view presented has important implications for the development of new therapeutic strategies for PH1 based on targeting specific elements of AGT protein homeostasis (summarized in Figure 3).

### 3.1. Stability of AGT Variants toward Chemical Denaturants, Temperature, and Proteases: Mechanistic Implications

Several studies have addressed the unfolding of AGT, both wild type (WT) and PH1 mutants, by either chemical denaturants (guanidine, urea, and pH) [55–57] or temperature [44, 48, 56, 57]. The mechanistic studies derived from these studies are discussed in this section in some detail. 

#### 3.1.1. Chemical Denaturation of AGT WT and Disease Causing Variants: Presence of Unfolding Intermediates

 Chemical unfolding by guanidium, urea, and mild acidic pH has been shown to irreversibly denature AGT at the experimental conditions used by different research groups [55–57]. We must note that the term *irreversible* is used here to indicate that removal of the denaturant does not provide the refolding yields required for applying equilibrium thermodynamic analyses. However, we must also note that very mild refolding conditions have proved to enhance remarkably AGT refolding yields [58]. Therefore, we will not attempt to extract thermodynamic information from the biophysical studies discussed here, even though they may provide insight into partially folded states that could be relevant to understand the (un)folding pathways of AGT variants *in vitro* (and possibly intracellularly), the effect of PH1 mutants on folding of AGT and the role of molecular chaperones in AGT folding and misfolding.

**Figure 4:**
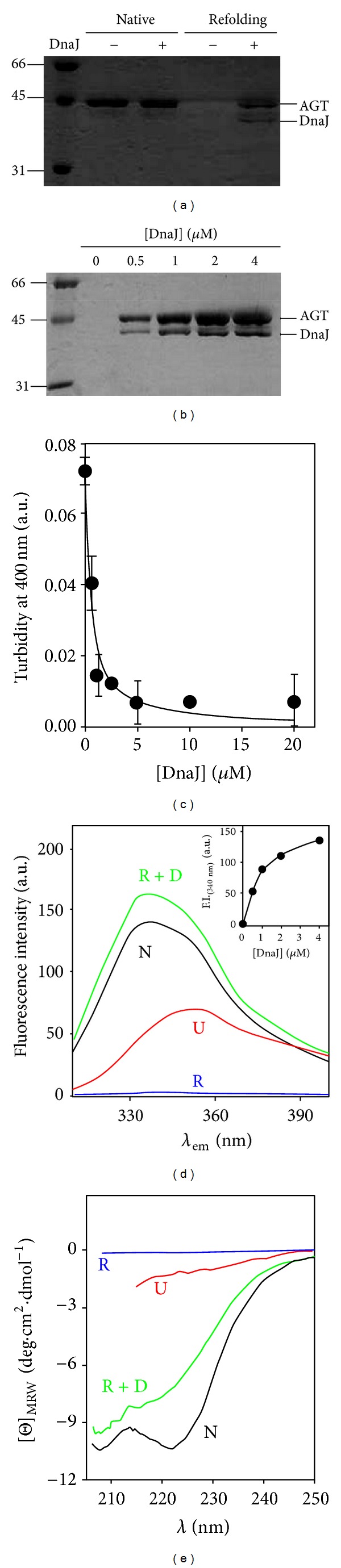
DnaJ prevents aggregation of a partially folded state of AGT. (a) IMAC-based copurification assay of his-tagged apo-AGTwt with DnaJ (1 *μ*M each), under native (no urea) and refolding (20-fold dilution from a 16 h incubated sample with 8 M urea) conditions; (b) DnaJ protein concentration dependence of its interaction with apo-AGTwt under refolding conditions (the same conditions as in (a)). (c) DnaJ protein concentration dependence of the maximal turbidity at 400 nm in the apo-AGTwt refolding (data are from three independent experiments); (d) and (e) solubility and conformational assays of Apo-AGTwt based on its intrinsic Trp-emission fluorescence ((d); exc.295 nm) or Far-UV CD (e) under different conditions: native (0 M urea; N), unfolded (8 M urea; U), and apo-AGTwt *refolded* in the absence (R) or presence of 4 *μ*M DnaJ (R + D). After urea-dilution, samples were incubated at 25°C for 30 min, centrifuged at 15000 rpm for 30 min, and the spectroscopic analyses were performed in the supernatants. The contribution from DnaJ to fluorescence is negligible due to the absence of Trp residues, while its contribution to Far-UV-CD spectra was subtracted from R + D. Inset: DnaJ protein concentration dependence of the Trp-fluorescence on the soluble fraction. A fitting to a hyperbolic function is shown, providing half-maximal fluorescence recovery at 1.0 ± 0.1 *μ*M DnaJ. All the experiments were performed at 25°C in Na-Hepes 20 mM NaCl 200 mM pH 7.4 2 mM DTT using 1 *μ*M AGT (in protein subunit). DnaJ was purified according to [4].

Unfolding of holo-and-apo AGT WT, minor AGT (LM, p.P11L-I340M) and LM-G170R (p.G170R in minor AGT - LM-, LRM for short) variants by guanidium hydrochloride and mild acidic pH have revealed the presence of a molten-globule-like unfolding intermediate (MG). This MG intermediate does not refold to the native state spontaneously and is able to interact with molecular chaperones in cell-free systems, suggesting that last folding steps of AGT may require help from molecular *chaperones in vitro* and intracellularly [57]. Interestingly, Hopper et al. [56] have shown that native state ligands such as PLP and AOA stabilize AGT WT and LM towards guanidium denaturation, even though the thermodynamic or kinetic basis of such stabilization and their relation with the partially unfolded states are unclear. Aggregation-prone unfolding intermediates are also observed in the urea induced denaturation of AGT, especially in the apo-forms and/or when the P11L polymorphism is present [55]. Indeed, the partially unfolded states found in the unfolding pathways of LRM variant might be related to its tendency for mitochondrial import [55], also explaining the strong interaction of this variant along its folding process with Hsp70 and Hsp90 chaperones in cell-free systems [57]. Additionally, aggregation of apo-AGT WT in refolding trials is prevented by the presence of the bacterial Hsp40 DnaJ in a concentration dependent manner (Figure 4), suggesting that binding to this chaperone is the first step to engage the Hsp70 machinery (Figure 3). 

#### 3.1.2. Thermal Denaturation of AGT Variants: The Role of AGT Kinetic Stability in PH1

 Thermal denaturation of AGT variants has been monitored by multiple techniques, including activity, circular dichroism, differential scanning fluorimetry (DSF), and differential scanning calorimetry (DSC) [43, 44, 48, 55–57, 59, 60]. All these studies have shown that withdrawal of PLP has a dramatic effect in terms of stability, reducing 20–25°C AGT thermal stability. The denaturation of holoproteins is consistent with a single denaturation transition using multiple probes, while denaturation of apoproteins is sometimes described by a one or two thermal transitions, depending on the AGT variant and the technique monitoring denaturation [48, 55–57, 59, 60]. The presence of two denaturation events has been discussed as the uncoupled denaturation of the large and small domains [59]. However, we must note that enzyme inactivation coincides with the low *T*_*m*_ transition found by Far-UV CD and DSF [48, 56, 59, 60] and the single transition found by DSC [48, 57]. Moreover, kinetic analyses of DSC transitions based on a two-state denaturation model provide denaturation rates at 37°C for apoproteins in excellent agreement with those obtained from inactivation kinetics, indicating that DSC based kinetic analyses monitor irreversible denaturation and inactivation of apo-proteins [48]. Almost all studies coincide in the destabilizing effect of the minor allele, while additional mutations further reduce AGT thermal stability [44, 48, 56, 57, 59, 60]. 

We have recently performed a thorough characterization of thermal denaturation of holo- and apoproteins by differential scanning calorimetry in AGT WT and eleven mutants and polymorphic variants found in PH1 patients [48, 57]. Denaturation of all AGT variants as apo- and holoproteins is described by a phenomenological two-state irreversible denaturation model. In this scenario, the stability of the native state is determined by the rate of irreversible denaturation *k*, that relates to the half-life for denaturation by *t*_1/2_ = ln2/*k* [2, 48, 57]. The value of *k* is determined by the height of the activation free energy that the native state must cross to reach the denaturation (rate-limiting) transition state (TS) [48, 57, 61]. Thermal denaturation of AGT mostly involves a dimeric TS, indicating that the kinetic stability of AGT enzymes is mainly dictated by the impact of mutations on the free energy of the dimeric native and TS states. Thus, dimer dissociation and monomer unfolding do not contribute to the AGT kinetic stability, because these steps must occur after the denaturation rate-limiting step [48]. The large kinetic stabilization exerted by PLP results from its preferential binding to the native state, increasing the denaturation free energy barrier by ~7 kcal·moL^−1^ [48, 57]. The temperature dependence of calorimetric enthalpies agrees well with the theoretical value for a dimer of this size, suggesting that holo- and apo-proteins unfold extensively and to a similar extent upon thermal denaturation, and possibly involving denaturation of both domains in AGT [48, 57].

Kinetic analyses of DSC traces provide a unique opportunity to compare denaturation rates among AGT variants spanning a wide range of stabilities (half-lives from minutes to years at physiological temperature). For instance, removal of PLP decreases (in WT and most of PH1 variants) AGT kinetic stability by 4-5 orders of magnitude [48, 57]. The presence of P11L and LM polymorphism reduces AGT kinetic stability by ~150 and ~20-fold, while the presence of I340 M kinetically stabilizes AGT compared to AGT WT (Fabelo-Rosa, submitted). In general, PH1 causing mutants show similar stability to AGT LM as holo-proteins, with a remarkably high kinetic stability (half-lives in the range of years at 37°C), while they often reduce apo-AGT kinetic stability (and denature in a time scale of minutes-hours; [48, 57, 62]). Thus, PLP binding overstabilizes the AGT native state in some mutants [2] which might explain the PLP responsiveness found for some of them in PH1 patients [63, 64]. Therefore, targeting the cellular systems responsible for PLP bioavailability (enzymes involved in PLP recycling and delivery; [65]) may represent a pharmacological approach to overcome mutation induced protein destabilization in PH1.

#### 3.1.3. Resistance to Proteolysis: Effects of PH1 Mutations on Protein Flexibility and High-Energy States

**Figure 5:**
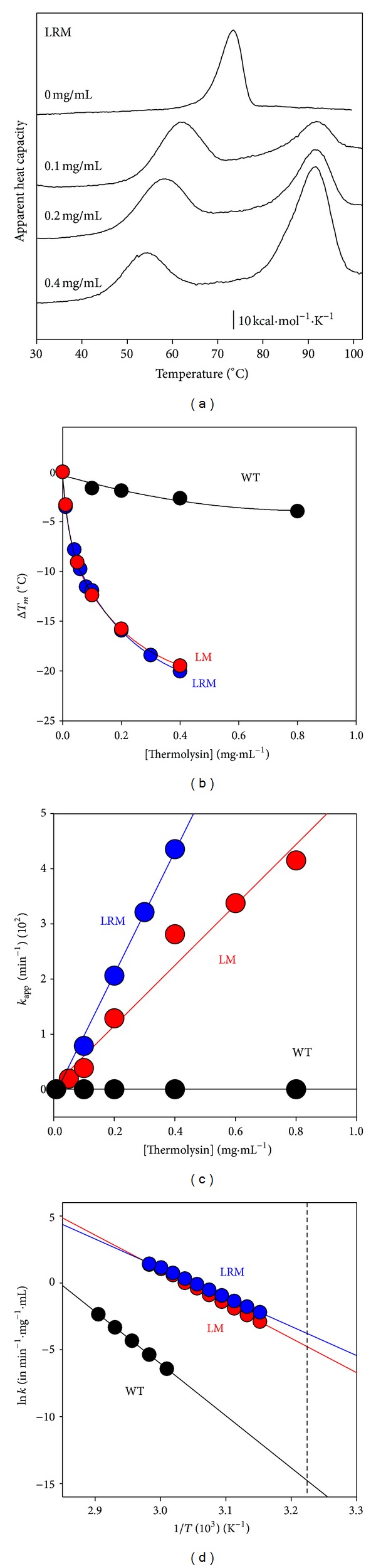
Temperature dependent proteolysis of AGT variants by thermolysin. (a) thermal scans of AGT-LRM (10 *μ*M in protein subunit) in the presence of different thermolysin concentrations (0–0.4 mg/mL). The high temperature transition (*T*_*m*_ ~ 90°C) corresponds to thermolysin denaturation, which is thermostable. (b) Dependence of *T*_*m*_ downshift of thermal transitions of AGT variants by thermolysin. (c) Dependence of the apparent proteolysis rate constants on thermolysin concentration at 44°C. The slope of these plots provides the second-order rate constants (plotted in (d)); (d) Arrhenius plots for the second-order rate constants for proteolysis. The vertical dashed line indicates 37°C. Experiments were performed in Hepes 20 mM NaCl 200 mM CaCl_2_ 10 mM.

 Proteolysis is an excellent method to study protein flexibility and transient population of protein high energy states, as long as flexible or partially unfolded regions in the protein are accessible for binding and proteolytic cleavage [66]. Indeed, kinetics of proteolysis has provided information on protein high energy states present in native state ensembles which are not accessible for most of ensemble averaged-based biophysical methods [66]. Several studies have addressed the stability of AGT enzymes towards proteolysis using different proteases ([34, 43, 57, 67, 68] and Figure 5). Trypsin and proteinase K degradation have been applied to several PH1 mutants [34, 43, 68], showing that P11L polymorphism enhances protease sensitivity that is further exacerbated by additional mutations. In the case of LM-G41R, detailed characterization of proteolysis products in combination with molecular dynamic simulations supports that G41R mutation enhances conformational fluctuations in the N-terminal region of AGT, thus accelerating cleavage by proteases [43, 68]. Interestingly, native state ligands and naturally occurring osmolytes are able to increase protease resistance in several PH1 mutants [68], suggesting that reshaping of the native state ensemble energetics by ligands may modulate the conformational fluctuations in some PH1 mutants. From a physiopathological viewpoint, studies in cell free systems on the proteasomal susceptibility also reveal that disease causing mutations enhance degradation [67]. 

AGT WT undergoes rapid cleavage by thermolysin leading to an active ~75 kDa dimer (referred here to as D* state) [57]. The stability of this D* state has been further characterized by a combination of thermolysin digestion and thermal scans for different holo-AGT enzymes (Figure 4). The apparent rate constants for proteolysis are extracted from thermal scans at a given protease concentration in the temperature range of the transition [69]. The *T*_*m*_ value of LM and LM-G170R is shown to be more sensitive to the concentration of protease than that of WT AGT (Figure 4(b)).The apparent first-order rate constants for proteolysis (Figure 4(c)) indicate that proteolysis is ~6 orders of magnitude slower for WT AGT than for the LM and LM-G170R, while LM-G170R is degraded about twice faster than AGT LM. At 37°C (Figure 4(d)), the half-lives against proteolysis (at 0.1 mg/mL protease) are 60-(WT), 3.9*·*10^4^-(LM), and 6.2·10^4^-fold (LM-G170R) lower than those determined in the absence of protease, suggesting that holo-AGT enzymes might be very sensitive to proteolysis despite their robustness towards thermal induced aggregation.

### 3.2. Protein Homeostasis of AGT: Implications to Develop New Therapeutic Strategies

 Peroxisomal import of human AGT occurs upon direct interaction of the fully folded dimer with Pex5p in the cytosol. Multiple lines of evidence support that protein folding defects are implicated in AGT loss of function in PH1 [30, 48]. However, dissecting the protein homeostasis defects in PH1 represents a remarkable challenge, since the biomolecular interactions of partially folded states with the protein homeostasis network may involve over 200 different proteins in cytosolic folding ([53]; see Figure 3 for a very *simplistic* view in PH1). In this section, we do our best to gather the most relevant available information on the physiological folding and peroxisomal targeting, as well as those protein homeostasis defects alterations that may lead to AGT loss of function due to different mechanisms, including mitochondrial mistargeting, enhanced protein aggregation, and degradation.

#### 3.2.1. Peroxisomal and Mitochondrial Targeting of Human AGT

 The PTS1 of human AGT is suboptimal (-KKL versus the consensus-SKL; [41]), and its molecular recognition by Pex5 requires additional structural elements at the C-terminal domain [29, 30]. In fact, the interaction of AGT WT and Pex5p PTS1 binding domain (Pex5p-pbd) is of moderate affinity (*K*_*d*_ ~ 1.5–3.5 *μ*M; [30, 48]) and decreases 10-fold for the isolated C-terminal PTS1 octapeptide [2, 28]. The low affinity of human AGT for its peroxisomal receptor may have several important implications to understand the evolutionary changes in AGT subcellular location and function [70, 71], the correct peroxisomal biogenesis [28], and the role of peroxisomal import in PH1 pathogenesis [30, 48].

 AGT subcellular location seems to vary among species depending on a delicate balance between mitochondrial and peroxisomal import signaling pathways and the capacity of cytosolic protein homeostasis networks to fold AGT proteins [70, 71]. The presence of mitochondrial AGT in carnivores, peroxisomal AGT in herbivores, and both locations in omnivores suggest that subcellular location of AGT may have resulted from dietary selection pressure [72]. Additionally, the presence of a strong N-terminal mitochondrial targeting sequence (MTS) in AGT from *X. laevis* drives the mitochondrial localization of this enzyme, while the corresponding N-terminal MTS seems to be weak in human AGT [71]. The weak MTS in hAGT seems to be strenghtened by additional natural variations associated with mitochondrial mistargeting, such as P11L polymorphism and the G170R, F152I and G41R mutations [38, 39, 73, 74]. Only the LM-G41R seems to be N-terminal cleaved upon mitochondrial import [38, 73], possibly because of the enhanced conformational fluctuations caused by the mutation next to the MTS [43], which suggests that the MTS is not generally cleaved upon import of hAGT as observed for many other MTS-containing proteins. Interestingly, proteins containing short MTS are known to be imported by a mechanism where unfolding is rate-limiting [75, 76], and thus, the low kinetic stability of LM-G170R, LM-F152I and LM-G41R as apo-proteins [43, 48, 57, 62] may speed up mitochondrial import of these mutants.

Besides the classical interaction between TOM20 mitochondrial receptors and N-terminal MTS, alternative mechanisms of mitochondrial import might be involved, such as presentation of internal targeting sequences in partially folded states to TOM20 or TOM70 receptors by molecular chaperones ([57, 77, 78]; see Figure 3). Enhanced interaction of misfolded/partially folded PH1 variants with molecular chaperones seems to a common feature to many PH1 mutants [34, 48, 50, 57], and thus not only restricted to the mistargeting LM-F152I and LM-G170R variants. Some mutations, such as LM-I244T, have been studied by several groups using different cell lines (COS and CHO) for heterologous gene expression, different cell culture media and PLP concentrations. Consequently, significant differences in mutant outcomes have been observed, showing either peroxisomal aggregation [34] or mitochondrial mistargeting [73], further supporting the view that different AGT *load* and variable capabilities of protein homeostasis networks may have a large impact on the final fate of PH1 causing mutants. Dissection the molecular details of these protein homeostasis defects is thus required to develop specific therapeutic strategies targeting different PH1 molecular mechanisms, possibly involving the cooperation of multiple proteostasis elements (Figure 3) including Hsp60, Hsp70 and Hsp90 machineries [34, 48, 50, 57]. 

 A recent study on PTS1 octapeptides from 42 human peroxisomal proteins has shown that these sequences range over four orders of magnitude on their affinity for Pex5p-pbd, and interestingly, the PTS1 corresponding to hAGT is the second weakest among this list [28]. A detailed analysis on protein expression levels for these peroxisomal proteins revealed a remarkable negative correlation between binding affinities and expression levels of cargo proteins, thus providing a mechanistic framework to generating a uniform population of Pex5p-cargo complexes necessary for proper peroxisomal biogenesis [28]. Pex5p-pbd binding studies of PH1 causing mutations G170R and V336D on the major allele [30] as well as some of the most common mutations on the minor allele, including G170R, I244T and F152I [48], revealed no changes in the molecular recognition of PH1 mutants by Pex5p, further supporting that folding defects are likely responsible for mitochondrial mistargeting and protein aggregation mechanisms in PH1. 

#### 3.2.2. Folding Defects Are Common to Mitochondrial Mistargeting and Aggregation in PH1: Targeting Protein Homeostasis Networks as Pharmacological Strategies for PH1

 Early studies in liver samples from PH1 patients showed that the LM-G170R variant reduces the protein levels and activity and lead to mitochondrial mistargeting [39]. Further studies, expanded to LM-G41R/LM-F152I compound heterozygotes showed combined mitochondrial/peroxisomal location, intraperoxisomal aggregation and null activity [38]. The LM-F152I, LM-G170R and LM-I244T variants show reduced folding efficiency in both prokaryotic and eukaryotic systems [40, 48, 57, 73], while a milder effect is observed for LM polymorphic variant in these studies. These evidences clearly pointed to PH1 as conformational disease. However, up to date, the molecular details of PH1 protein homeostasis defects are mostly unknown.

 We have recently initiated a comparative study on several PH1 variants that shows a positive correlation between apo-AGT kinetic stability, interaction with molecular chaperones, and decrease solubility and total protein levels in transiently transfected CHO cells [48]. This seems to apply for either aggregation (LM-I244T) or mistargeting (LM-F152I and LM-G170R) mechanisms. Accordingly, a recent study has shown that human AGT is capable of complementing yeast strains lacking endogenous yeast AGT in glycine-free medium [56], while LM-F152I and specially LM-I244T, but not LM-G170R, reduced the complementation elicited by AGT LM. Complementation by PH1 mutants in this system closely correlated with steady-state expression levels, indicating that these mutants affect proper intracellular folding *in vivo*. More recently, using a novel stability reporter assay [60], a clear correlation between *in vivo* steady AGT levels and kinetic stability and yeast growth was found for LM-F152I, LM-G170R and LM-I244T. Overall, these expression studies support a link between intracellular folding efficiency and kinetic stability of apo-AGT variants associated to mistargeting and aggregation mechanisms. 

As we have mentioned in Section 3.2.1, the intracellular fate of AGT mutants depends on the experimental conditions of *in vitro* expression (LM-I244T is an excellent example; [34, 48, 73]). An interesting possibility to explain these differences is that different capacities of the protein homeostasis networks might determine the phenotype at the molecular level (aggregation versus mistargeting), a phenomena that has been described for disease penetrance even among isogenic individuals in animal models of several folding diseases [79]. These differences in folding capability might explain different phenotypic outcomes even among siblings sharing a given phenotype [2], and also possibly, the different response to pyridoxine supplementation among PH1 patients with the same phenotype [63, 64].

#### 3.2.3. Interaction with Molecular Chaperones

Protein folding intermediates are commonly observed for proteins larger that 100 aminoacids, and these intermediates often require substantial structural reorganization upon interaction with molecular chaperones to reach the native conformation [80]. As we describe in Section 3.1.1, human AGT is known to populate (un)folding intermediates upon chemical denaturation, and these intermediates do not generally reactivate [55, 57] and also stably interact with several types of molecular chaperones ([34, 50, 57]; Figure 3). Moreover, the strong interaction of several misfolding AGT mutants with molecular chaperones suggests that these chaperones might be important checkpoints in AGT folding and misfolding [34, 50, 57]. Thus, modulation of the interaction of partially folded states of AGT variants with molecular chaperones is a plausible approach for pharmacological intervention in PH1, as similarly described for other protein folding diseases [81–83]. 

 An important question is whether WT and PH1 causing mutants populate different folding/unfolding intermediates, or whether, kinetic/thermodynamic aspects of these folding intermediates and their interaction with the protein homeostasis networks [84–86] determine the final fate of AGT variants (mistargeting versus aggregation versus degradation). The structure/energetics of the TS for the rate limiting step of irreversible denaturation of WT and PH1 mutants is strikingly similar, suggesting a fine-tuning of the structure/energetics of this TS as determinant for the kinetic stability of PH1 mutants (unpublished observations). On the other hand, the structural properties of the chaperone competent MG state formed at mild acidic pH is similar for WT, LM and LM-G170R, but seems to accumulate in a Hsc70- or Hsp90-bound state upon expression in cell-free systems for LM-G170R [57]. Moreover, aggregation of AGT WT is prevented *in vitro* by its interaction along its folding pathway with Hsp40 in a partially folded state that resembles the acid-induced MG state (Figures 4(d)-4(e)), which may prevent AGT aggregation co- or post-translationally, thus delivering the polypeptide to the Hsc70 machinery [80, 86]. Hsc70 machinery may represent the first essential folding checkpoint, as the interaction of these chaperones with Hsp40 proteins and nucleotide exchange factors [86] would determine the partitioning between correct folding and peroxisomal import, mitochondrial import or degradation (Figure 3). Transfer of cargo AGT from Hsp70 to Hsp90 might also occur through the Hsc70-Hsp90 organizing protein (HOP; [86]). Moreover, we have also described that overexpression of bacterial Hsp60 (GroEL; [50]) increase the recovery of LM-I244T in *E. coli*, forming stable complexes with GroEL. The characterization of the bound AGT LM-I244T by cryoelectron microscopy shows that AGT monomers display folded N- and C-terminal domains in an open conformation [50]. Overall, these studies suggest that the last steps in AGT folding involve domain docking and acquisition of quaternary structure that are crucial for AGT conformation of PH1 mutant. Thus, pharmacological modulation of the proteostasis pathways involved may rescue AGT function in PH1 patients.

## 4. AGT as a Drugable Target for the Treatment of PH1

Once the diagnosis of PH1 is made, or even suspected, conservative measures should be initiated as soon as possible with the goal of preserving renal function. Once renal function is lost, the threat posed by progressive oxalate accumulation makes it necessary to perform liver and kidney transplantation, an aggressive treatment not free of difficulties, risks and limitations. Thus, current research aims at medical treatments for PH1.

High fluid intake has been proven to be effective in kidney stone diseases [87]. In PH, the recommended fluid intake is at least 3 L/m^2^ per day, and special care should be taken in situations of fluid losses (diarrhea, vomiting, and fever) or limited oral hydration (surgery), where i.v. fluid administration might be necessary to keep high urine flow. In addition, alkalinization of the urine with alkali citrate is implemented to reduce urinary CaO*x* saturation.

Administration of pyridoxine hydrochloride was proposed as a conservative PH1-specific measure several decades ago. This form of treatment has been known to be associated with a decrease in urinary oxalate (UO*x*) in about 30% of PH1 patients [88, 89]. Since vitamin B6 is very safe, with a small risk of sensory neurotoxicity as the main side effect, a trial of pyridoxine treatment should be performed in most cases, and particularly in patients with missense mutations. If responsive, patients should be treated until liver transplantation is performed, even if they are in ESRD undergoing haemodialysis. The recommended starting dose is 5 mg/kg per day, increasing in 5 mg/kg steps to a maximum of 20 mg/kg body weight per day [90]. Responsiveness is currently defined by >30% decrease in UO*x* excretion after a test period of a minimum of 3 months at maximum dose [22, 91]. A subset of patients carrying one or two copies of G170R or F152I mutations have been shown to respond best to pyridoxine [63, 64].

In addition to the experimental data obtained in the last few years on the cellular and molecular consequences of various missense mutations, the empirical response of some PH1 patients to vitamin B6 treatment supports the view of AGT as a “drugable target” that deserves serious commitment to develop small molecules with enhanced chaperone capability or proteostatic effect.

As we describe in detail in Section 3.1.2, binding of PLP to AGT protein dramatically enhances its kinetic stability, particularly in variants of the minor haplotype, such as G170R and I244T [57]. The most relevant parameter to estimate the kinetic stability of properly folded cytosolic AGT dimers seems to be the half-life for irreversible denaturation of the apo-forms. Conversion of apo- to holo-AGT is a slow process that is catalyzed and regulated by specific enzyme systems [57, 65], and this conversion may trap the AGT in a folded and kinetically stable holo-form ready for peroxisomal import. This is especially relevant for PH1 missense mutations that are more destabilized in their apo-form than in their holo-form. 

Building on this view of PLP as a natural kinetic stabilizer of AGT, it is reasonable to expect that pharmacological chaperones will be found that specifically bind to the AGT native state leading to thermodynamic and/or kinetic stabilizations. Ideally, they will have enhanced chaperone activity on mutant AGT variants, leading to more efficient conservative treatments for PH1. To move forward, a more precise undestanding of the mechanism of PLP kinetic “overstabilization” of missense mutations is needed. The precise molecular details of this overstabilization are under investigation.

Nevertheless, since protein stabilization can be searched by high-throughput screening of chemical and virtual libraries [92, 93], the possibility exists that potential pharmacological chaperones are found empirically. In some cases, pharmacological chaperones resemble known protein ligands or inhibitors, and their stabilizing effect may be enhanced by structure-based approaches [93, 94]. Pharmacological chaperones have been shown to correct protein misfolding in several genetic diseases and it seems likely that missense PH1 mutations can benefit from this approach also. In addition, targeting cellular systems responsible for PLP bioavailability (enzymes involved in PLP recycling and delivery; [65]) may represent a pharmacological approach to overcome mutation induced protein destabilization in PH1.

Chemical chaperones are small organic compounds which favor compact protein states over unfolded states through the so-called “solvophobic effect”, which involves destabilizing interactions of the water/chaperone mixture with the polypeptide backbone [95]. Their beneficial effect on mutant AGT has been demonstrated *in vitro* [34, 43], although the high concentrations required for protein stabilization and the lack of specificity of the stabilizing effect make them poor candidates to move forward into preclinical investigation.

In the future, the complexity of AGT intracellular homeostasis will also be better understood. The macromolecular assemblies that assist AGT expression, folding, stability, intracellular trafficking and degradation include key components that are also drugable targets for conservative treatment of PH1. AGT native state kinetic stability [57, 62] and interactions of partially folded states with molecular chaperones such as Hsp40/DnaJ, Hsp60/GroEL, Hsc70 and Hsp90 [34, 48, 50, 57] may be involved in the partition of AGT protein between correctly folded dimers and misfolding, with mitochondrial mistargeting, aggregation and degradation.

Pharmacological modulation of molecular chaperones is a promising therapeutic strategy in several conformational diseases [82]. However, the complexity of the molecular chaperone system in humans poses a significant challenge. The HSP70 machinery, for instance, includes at least 11 HSP70s, 41 J-proteins and 13 nucleotide exchange factors (NEFs) [86]. In addition, mammalian J-proteins and NEF proteins are structurally and functionally complex, displaying additional functions beyond the canonical model. J-proteins are known to be involved in shunting client proteins for degradation, remodeling and partially unfolding client proteins, while the BAG (BCL2-associated athanogene) family of NEFs are also known to target client proteins for proteasomal degradation [86]. Thus, finding the ideal target and modulating it pharmacologically in just the right direction is a formidable challenge. As a first step, an exhaustive biochemical and cellular definition on all these targets is needed, in order to determine those responsible for mitochondrial mistargeting, aggregation and degradation of mutant AGT.
